# Reply to F. Tomao et al

**DOI:** 10.1200/JCO.2016.69.4158

**Published:** 2016-09-12

**Authors:** Karin Ribi, Jürg Bernhard, Weixiu Luo, Meredith M. Regan, Gini F. Fleming, Prudence A. Francis

**Affiliations:** Karin Ribi, International Breast Cancer Study Group Coordinating Center, Bern, Switzerland; Jürg Bernhard, International Breast Cancer Study Group Coordinating Center; and Bern University Hospital, Inselspital, Bern, Switzerland; Weixiu Luo, International Breast Cancer Study Group Statistical Center, Dana-Farber Cancer Institute, Boston, MA; Meredith M. Regan, International Breast Cancer Study Group Statistical Center, Dana-Farber Cancer Institute; and Harvard Medical School, Boston, MA; Gini F. Fleming, The University of Chicago Medical Center and Alliance for Clinical Trials in Oncology, Chicago, IL; and Prudence A. Francis, Peter MacCallum Cancer Center, St Vincent’s Hospital, University of Melbourne, Melbourne; and University of Newcastle, Newcastle, NSW, Australia

We thank Tomao et al^1^ for their comments on our recent report on patient-reported outcomes (PROs) in the Suppression of Ovarian Function Suppression Trial (SOFT).^2^ When SOFT was designed more than a decade ago to investigate the role of ovarian function suppression (OFS) and the role of exemestane in premenopausal women with endocrine-responsive early breast cancer either after completion of (neo)adjuvant chemotherapy or after surgery alone,^3^ quality of life (QoL) was integrated to assess the patients’ perspective. Our results provide complementary information to the adverse event reporting in SOFT^2^ and add novel information to existing evidence on the effect of OFS on PROs and QoL. In the Zoladex in Premenopausal Patients^4,5^ trial, chemotherapy was given concurrently with adjuvant endocrine therapy, and the observation period was restricted to the first 2 years of treatment. Eastern Cooperative Oncology Group E-3193^6^ and SOFT are the only two randomized phase III trials presenting long-term PROs in this setting. Our article complements the findings of E-3193, but with major differences. The SOFT population is international, and the sample size is larger, with 1,722 patients available for the primary QoL analysis. The E-3193 trial presented data from a summary score of patient-reported symptoms. We found that OFS added to tamoxifen results in variable magnitudes of treatment differences for individual symptoms. The E-3193 trial only included patients without chemotherapy, whereas SOFT enrolled two distinct cohorts of patients: those with and those without prior chemotherapy. The prior chemotherapy cohort had higher-risk disease than the E-3193 trial population, which is the current target population for OFS. Cognitive function was assessed as part of a substudy.^7^ The gold standard of cognitive assessment, a comprehensive neuropsychological testing,^8^ was not feasible in the entire study population, with more than 500 centers and many different languages.

Tomao et al^1^ suggest that we did not present several clinically relevant data. With regard to treatment, in the cohort of patients with prior chemotherapy, the majority received either anthracycline-based (38%), or anthracycline plus taxane–based (53%) chemotherapy. The impact of these two different types of chemotherapy on patients’ symptom experience is relevant primarily in the short term. The number of patients who were treated with human epidermal growth factor receptor 2–directed therapy (ie, trastuzumab, 8% overall) were reported in Table A1 (Data Supplement) of Ribi et al.^2^ This low percentage would not have changed our results in a meaningful way. Oral endocrine therapy before randomization was allowed while premenopausal status was established or re-established. Details on duration were included in our report (Table 1).^2^ In the overall population, three patients received an aromatase inhibitor before randomization.^3^ The number of patients with irregular menstruation or persistent amenorrhea at baseline (ie, after chemotherapy or previous endocrine therapy) were reported in Table A1 in the data supplement.^2^ We controlled for menstruation status in the mixed-effect models. During the 5 years of assigned endocrine treatment, it was not possible to accurately determine menopausal status, as we did not routinely measure hormone levels during treatment and amenorrhea during tamoxifen is not an accurate determinant of menopause.

A further criticism relates to the inclusion of patients who both did and did not receive chemotherapy before enrollment. The authors are correct that patients in the chemotherapy cohort had higher-risk disease characteristics and were younger (Table 1 in our report and Table A1 in the Data Supplement).^2^ However, the occurrence of symptoms (eg, vasomotor, gynecologic, and sexual symptoms) was not lower in this cohort. On the contrary, patients with prior chemotherapy reported worse baseline scores for these symptoms compared with the no-chemotherapy cohort, possibly caused by chemotherapy and by the receipt of tamoxifen before enrollment in half of these patients. Thus, changes over time in these symptoms were smaller (ie, less worsening) in the chemotherapy compared with the no-chemotherapy cohort, leading to the interpretation that chemotherapy did not exacerbate adverse effects. Presenting our results not only for the overall population but also separately for the two chemotherapy cohorts in our report (Appendix Figs A2A and A2B)^2^ is a strength of our study.

The efficacy results from SOFT,^3^ in conjunction with those from the SOFT plus TEXT combined analysis,^9^ are practice changing.^10^ PROs comparing exemestane versus tamoxifen in patients who received OFS were published earlier this year.^11^ The PROs of the comparison of tamoxifen plus OFS versus tamoxifen alone for the cohorts with and without chemotherapy provide physicians and patients with a comprehensive picture of the risks and benefits when choosing the best adjuvant treatment for these relatively young women.
